# Seed dormancy, germination and storage behavior of *Magnolia sinica*, a plant species with extremely small populations of Magnoliaceae

**DOI:** 10.1016/j.pld.2021.06.009

**Published:** 2021-07-12

**Authors:** Liang Lin, Lei Cai, Lei Fan, Jun-Chao Ma, Xiang-Yun Yang, Xiao-Jian Hu

**Affiliations:** a The Germplasm Bank of Wild Species, Kunming Institute of Botany, Chinese Academy of Sciences, Kunming, 650201, Yunnan, China; b Yunnan Key Laboratory for Integrative Conservation of Plant Species with Extremely Small Populations, Key Laboratory for Plant Diversity and Biogeography of East Asia, Kunming Institute of Botany, Chinese Academy of Sciences, Kunming, 650201, Yunnan, China

**Keywords:** *Magnolia sinica*, Seed, Dormancy, Storage behavior, Cryopreservation, Excised embryo

## Abstract

*Magnolia sinica* is one of the most endangered Magnoliaceae species in China. Seed biology information concerning its long-term *ex situ* conservation and utilization is insufficient. This study investigated dormancy status, germination requirements and storage behavior of *M. sinica*. Freshly matured seeds germinated to ca. 86.5% at 25/15 °C but poorly at 30 °C; GA_3_ and moist chilling promoted germination significantly at 20 °C. Embryos grew at temperatures (alternating or constant) between 20 °C and 25 °C, but not at 5 °C or 30 °C. Our results indicate that *M. sinica* seeds possibly have non-deep simple morphophysiological dormancy (MPD). Seeds survived desiccation to 9.27% and 4.85% moisture content (MC) as well as a further 6-month storage at −20 °C and in liquid nitrogen, including recovery *in vitro* as excised embryos. The established protocol ensured that at least 58% of seedlings were obtained after both cold storage and cryopreservation. These results indicate that both conventional seed banking and cryopreservation have potential as long-term *ex situ* conservation methods, although further optimized approaches are recommended for this critically endangered magnolia species.

## Highlights

• Seeds of *Magnolia sinica* are probably non-deep morphophysiological dormant (MPD), not intermediate complex MPD (Baskin and Baskin, 2014).
• *Magnolia sinica* seeds exhibit orthodox storage behavior. This is the first report of orthodox storage behavior for a Chinese Magnolia species.
• Cryopreservation is also feasible for seeds of *Magnolia sinica*; however, further optimization for cryopreservation protocols is needed.

## 1 Introduction

Magnoliaceae is one of the families that dominates the subtropical evergreen broadleaved forest ecosystem in southwestern China (Li and Pritchard, 2009) and is also the most threatened Angiosperm family in China (Ministry of Environmental Protection of the People's Republic of China and Chinese Academy of Sciences, 2013). Many species of Magnoliaceae are critically endangered, including *Magnolia sinica* (Y.W. Law) Noot. In the *Flora of China*, *M. sinica* is listed as *Pachylarnax sinica* (Y.W. Law) N.H. Xia & C.Y. Wu (Xia et al., 2008), but *Manglietiastrum sinicum* Y.W. Law and its common name ‘hua gai mu’ are more well known amongst botanists and conservation practitioners. Because of its very limited population size and range, the species is classified as a plant species with extremely small populations (PSESP) (Ma et al., 2013; Wang et al., 2016). The species has been under threat from habitat degradation due to cutting and clearance. Until recently, limited protective measures have been put in place. Saplings have been available through various nurseries and these have been used to supplement the wild population. Now, both *in situ* and *ex situ* conservation measures have been implemented, including the establishment of protected areas, transplantation into botanical gardens and reintroduction of nursery-grown material to the wild (Yang et al., 2020). Whilst fresh seeds have been handled during these activities, the seed storage and germination biology of *Magnolia sinica* are little understood. In a recent review of the seed biology of PSESPs, it was only noted that species in this family tend to have orthodox (desiccation-tolerant) seeds (Wade et al., 2016). Thus, for the genus *Magnolia* (including species within *Manglietia*, which is a synonym of *Magnolia*), eight of the 11 species in the Seed Information Database have seeds that are likely to be orthodox (i.e., desiccation tolerant). The other three species have seeds that may not be desiccation tolerant (https://data.kew.org/sid/; accessed 4 Feb 2021). This variability in response of seeds of Chinese Magnoliaceae to drying has been noted as a problem before (Dent, 1948; Campbell, 1980; Lin and Huang, 1994; Han, 2008; Han and Long, 2010a, b; Han and Sun, 2013; Tang, 2014; Royal Botanic Gardens Kew, 2019).

If seeds of *Magnolia sinica* are desiccation tolerant, there should be the option of storing the seeds in a conventional seed bank, i.e., after drying to ca. 15% RH and transferring to −20 °C. This technology is the main means of conserving plant species *ex situ*, as a wide range of genetic diversity can be stored in a relatively small space (Li and Pritchard, 2009). However, the oily seeds/embryos of some species might survive drying but then lose viability in a few months to years when held at −20 °C; for example, *Coffea arabica* L. (Ellis et al., 1990) and *Elaeis guineensis* Jacq. (Ellis et al., 1991). As the oil content in *Magnolia* seeds is variable (49%, *Magnolia grandiflora* L.; 46%, *M. macrophylla* Michx., and 8%, *M. obovata* Thunb.; https://data.kew.org/sid/; accessed 4 Feb 2021), it is possible that such a storage response at −20 °C might also be a feature of seeds of some *Magnolia* species. Based on biophysical investigations of oily spores of ferns, the problem at −20 °C might relate to lipid crystallization over time; although this is not an issue when the material is cryopreserved (Ballesteros et al., 2019). Seeds and embryos of many species can tolerate cryopreservation, with potential benefits for longevity (Grout et al., 1983; Pritchard, 2007). Therefore, when seeds of threatened species are being committed to seed banks (Hong and Ellis, 1996), it is critical to assess desiccation tolerance and the impact of different low temperatures.

Of equal importance to understanding seed storage behavior in threatened species is knowledge about their seed germination traits. Quantification of the thermal and hydrological control of germination provides insights on plant regeneration potential and risks from climate change (Fernandez-Pascual et al., 2019). A geographical imprint on seed and seedling traits has been noted amongst fifteen Chinese provenances of *Magnolia officinalis* Rehder & E.H. Wilson, with nursery germination varying from 27 to 91% (Shu et al., 2012). In the case of *Magnolia grandiflora*, seed germination in a saran greenhouse increased ca. 10% after cold stratification for 90 days (Fetouh and Hassan, 2014). Similarly, moist chilling at 4 °C for 3 weeks can also effectively break the seed dormancy of *M. sinica* seed, with 56% of seeds treated in this way germinating after 30 days of incubation (Zheng and Sun, 2009). Similarly, after 100 days of cold stratification, seeds of *Magnolia wilsonii* (Finet & Gagnep.) Rehder germinated to 82% at 25/20 °C (Han and Long, 2010b). Moreover, 25/20 °C is known to be a suitable temperature for germination after dormancy alleviation by cold stratification or GA_3_ treatment of some other Magnoliaceae species, including *Magnolia duclouxii* (Finet & Gagnep.) Hu, *M. fulva* (Hung T. Chang & B.L. Chen) Figlar, *M. laevifolia* (Y.W. Law & Y.F. Wu) Noot. and *M. sphaerantha* (C.Y. Wu ex Y.W. Law & Y.F. Wu) Sima (Han, 2008).

The set of environmental conditions used to trigger germination and the basic seed morphology of the species can enable the categorization of seed dormancy (Baskin and Baskin, 2014). *Magnolia sinica* are thought to produce seeds that have intermediate complex morphophysiological dormancy (MPD) (Baskin and Baskin, 2014). This means that the seeds should have an embryo that does not fill the seed and needs to grow internally before germination; and the seed may require a pretreatment temperature (cold stratification) followed by exposure to a different temperature to maximize the germination level. However, according to our field observations, seeds can germinate without moist chilling, suggesting that dormancy state might not be intermediate complex MPD. To date, no information about the temperature effect on embryo growth is available for *M. sinica*, and this information is essential for the determination of its seed dormancy status.

Therefore, the purpose of this study on *Magnolia sinica* is to: 1) determine the seed dormancy status and temperature requirements for germination; 2) assess the level of desiccation tolerance and storage behavior of both seeds and embryos; and 3) investigate the effect of cryopreservation on seeds and embryos with different moisture contents. The findings have implications for both the conservation and utility of this magnolia species.

## 2 Materials and methods

### 2.1 Seed collection

Indehiscent fruits were collected form Jinping, Yunnan, China in November 2019, and transported within one week to the Germplasm Bank of Wild Species in Southwest China, Kunming Institute of Botany, Chinese Academy of Sciences. During transportation, the fruits opened naturally. On receipt in the laboratory, the seeds were taken out of the fruits by hands and the red, fleshy testa removed by rubbing against mesh under running water. Any excess moisture was removed by blotting seed surfaces dry.

### 2.2 Experimental design

#### 2.2.1 Seed anatomy and embryo growth

Seed anatomy was measured on 10 seeds using a Keyence VHX-6000 digital microscope (Keyence Co., Osaka, Japan). Both seed (with endotesta only) and endosperm length and width were measured. To determine the effect of temperature on embryo growth, seeds were incubated in light (12 h of 22.2 μmol m^−2^s^−1^ cool white fluorescent light and 12 h dark each day) at 5 °C for 13 weeks and the embryo and seed length were measured at the beginning and end of this incubation period. Thereafter, seeds were transferred to 20, 25, 30, 20/10 (light/dark), 25/15 (light/dark) and 30/20 °C (light/dark) and incubated for 4 weeks, with light applied as described above. Every 7 days at each temperature, 10 seeds removed and embryo dimensions were measured.

#### 2.2.2 Desiccation and storage

Seed initial moisture content (MC) was determined on 10 randomly-selected individual seeds by gravimetry after 17 h drying at 103 °C (ISTA, 1996). Seed desiccation was performed in a room operating at 15 °C and 15% relative humidity (RH). Based on the initial weight of the seed batch and known moisture content, the seeds were dried to two target MCs of around 10% and 5%. The first target MC (10%) was determined by monitoring the decrease in the sample weight every 2 h. When the sample weights reached target levels, the actual seed MC was determined by the method use for the initial MC. The second target MC (5%) was determined by detecting seeds equilibrium relative humidity (eRH) by a Rotronic HC2-AW probe attached to a Hygrolab C1 unit (Rotronic Ltd., Crawley, UK), when the eRH was around 15%, MC should be around 5%.

Seeds dried to the target MCs were sown for germination and the embryo excised and germinated *in vitro* (see details below). The seeds were stored −20 °C and in liquid nitrogen for 3 months. For cold storage at −20 °C, seeds were put into a hermetic jar; for cryopreservation, seeds were put into a cryovial and plunged into the liquid nitrogen tank. After cold storage at −20 °C, seeds were rewarmed in the dry room at 15 °C for 24 h and then germinated (see below). Seeds that had been cryopreserved were removed from liquid nitrogen and rewarmed in a water bath at 40 °C for 2 min before sowing for germination. After storage, seeds were sown for germination (see below) and embryos excised and germinated *in vitro* (see below).

#### 2.2.3 Germination test

For freshly matured seeds, three replicates of 20 seeds were sowed on 1% agar-water medium or 1% agar-water medium with 200 mg/L gibberellic acid (GA_3_) in 90 mm Petri dishes, and then incubated at 20, 25, 30, 20/10 (light/dark), 25/15 (light/dark) and 30/20 °C (light/dark); the photo period was 12 h (22.2 μmol m^−2^s^−1^ cool white fluorescent) light and 12 h dark each day.

A batch of freshly matured seeds were also cold stratified in light (12 h of 22.2 μmol m^−2^s^−1^ cool white fluorescent light and 12 h dark each day) at 5 °C for 13 weeks, thereafter transferred to the germination test as described above but only on 1% agar-water medium.

Before sowing for germination, the seeds dried to around 10% MC and around 15% eRH as well as seeds that stored for 3 and 6 months, were first pre-humidified above water at 20 °C for 24–48 h. Germination was at 25/15 °C on agar-water with 200 mg/LGA_3_ in 90 mm Petri dishes under conditions described earlier. Three replicates of 20 seeds were used for each treatment.

Germination was checked every 7 days, and a seed with a radical over 5 mm was considered to be germinated. Petri dishes were randomly rearranged on checking days. The germination test was run for at least 8 weeks. If no germination occurred for two consecutive weeks, the test was terminated. At the point of termination, un-germinated seeds were cut through to determine if they were empty, decayed or firm, and the total viability post-storage determined, i.e., germinated + firm seeds.

#### 2.2.4 In vitro *germination of excised embryos*

Seeds were surface disinfected by immersion in 75% ethanol for 2 min followed by 10 min in a solution of 0.5% Sodium dichloroisocyanurate with a drop of Tween-20. Under a laminar flow hood, the hard seed coat was cracked open with pliers and the zygotic embryo excised under a stereomicroscope. Embryos were incubated on 20 mL of Woody Plant Medium (Lloyd and McCown, 1980) with 0.5 mg/L 6-BA, 1 g/L activated charcoal, 30 g/L sucrose, and 3 g/L Phytagel. The 9 cm-diameter Petri dishes were incubated in the dark at 25 °C for 60 days.

### 2.3 Calculation and statistical analysis

Moisture content (MC) was determined on a fresh weight basis as:

MC = (FW-DW) / DW × 100%

where FW is the fresh weight and DW the dry weight of seeds.

The embryo/seed ratio (E:S) is calculated as:

E:S = Le/Ls × 100%

where Le is the embryo length and Ls is the length of the seed.

Germination percentage (GP) used for dormancy assessment was calculated as:

GP = Σni / (N-Ne-Nd) × 100%

Viability (V) was calculated as:

V = (N-Ne-Nd) / N × 100%

where N is total number of seeds tested, Ne number of empty seeds, Nd number of decayed seeds and ni number of germinated seeds.

The germination of excised embryos (GPe) was calculated as:

GPe = Ng / (Nt-Nc)

where Ng is the number of germinated embryos, Nt is the total number of embryos excised and Nc is the number of embryos contaminated.

For the embryo/seed ratio, seed gemination and viability data, means were compared by a one-way ANOVA for each treatment, and an LSD post hoc was applied for different treatments. Differences obtained at a level of *P* < 0.05 were considered to be significant. This analysis was performed by SPSS 16.0 for windows (SPSS, Chicago, IL, USA).

For germination data of excised embryos, a chi-square test was performed using R (R Core Team, 2019) between initial germination and all other treatments; differences obtained at a level of *P* < 0.05 were considered to be significant.

## 3 Results

### 3.1 General seed characteristics, seed anatomy and embryo growth

**Fig. 1:**
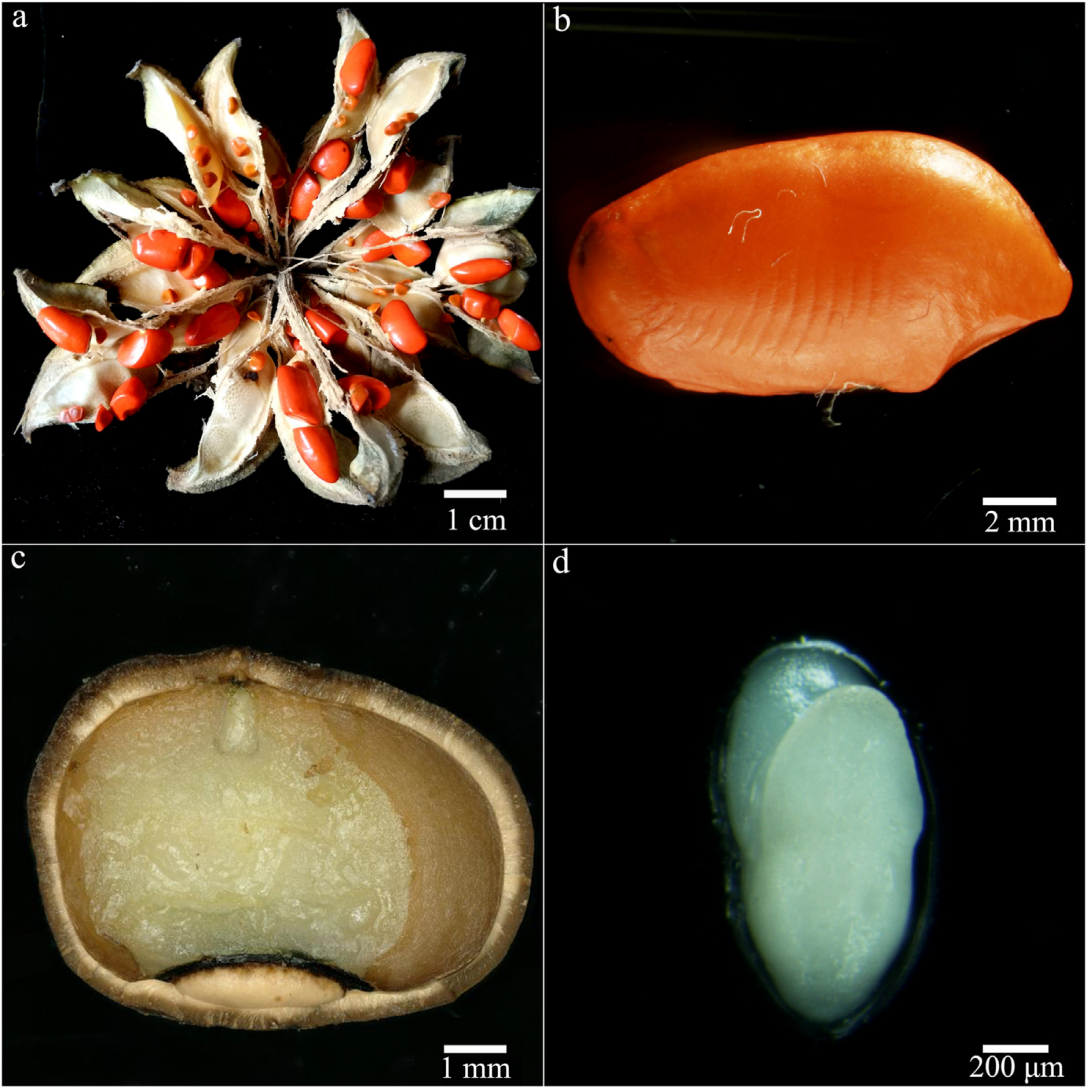
Fruit, seed and embryo morphology of *Magnolia sinica*. Dehiscent fruit (a), seed (b), profile of seed (c) and excised embryo (d).

**Fig. 2:**
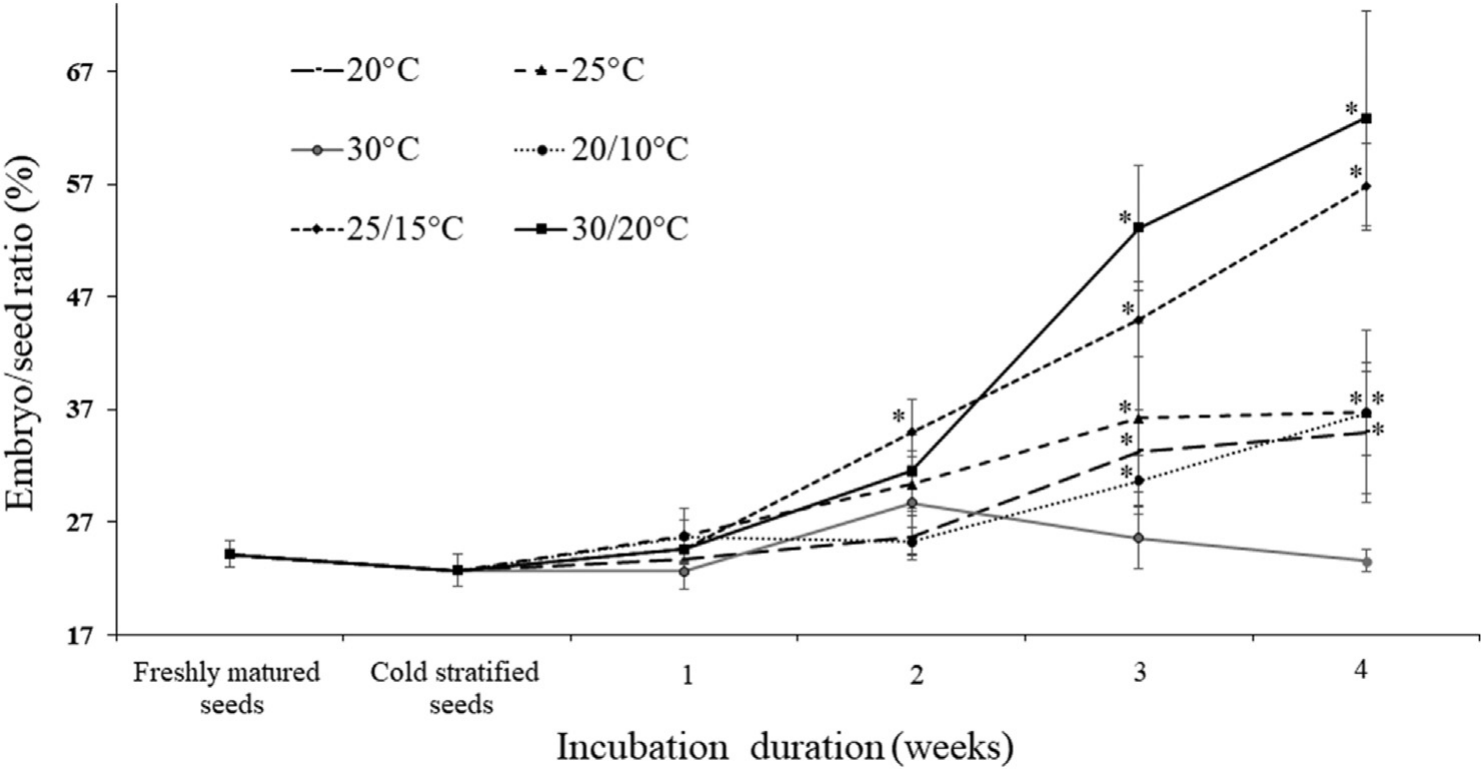
Embryo/seed ratio (mean ± s.e.) of freshly matured seeds of *Magnolia sinica* and seeds cold stratified for 13 weeks followed by up to four weeks at different temperatures. Asterisk (∗) indicates significant difference compared with the data of freshly matured seeds.

The testa of freshly matured seed was colored orange-red to red (Fig. 1a and b), and the bony endotesta was brown. Seed (without testa) was broad obovate in shape (Fig. 1c), 58.27 ± 7.34 mm long (from embryo side to hilum side) and 86.43 ± 8.69 mm wide. The endosperm was kidney shaped and abundant (Fig. 1c), 37.09 ± 3.78 mm long by 76.52 ± 7.56 mm wide. The thousand seed weight was ca. 77.62 g for dry (4.85% MC) seeds (without testa). Freshly matured seeds had small, rudimentary embryos 0.89 ± 0.14 mm long (Fig. 1c and d). Proportionally, this initial embryo length was 0.24 that of the seed (Fig. 2). During cold stratification for 13 weeks, the embryo/seed (E:S) ratio decreased a little bit, but not significantly. After moving the seeds from the cold to warmer temperatures, the E:S ratio increased at all temperatures (20, 25, 20/10, 25/15 and 30/20) except 30 °C. The internal growth of the embryo was fastest at 25/15 and 30/20 °C, about double to ca. 0.6 the length of the seed within 4 weeks (Fig. 2).

### 3.2 Germination of freshly matured seeds and after cold stratification

**Fig. 3:**
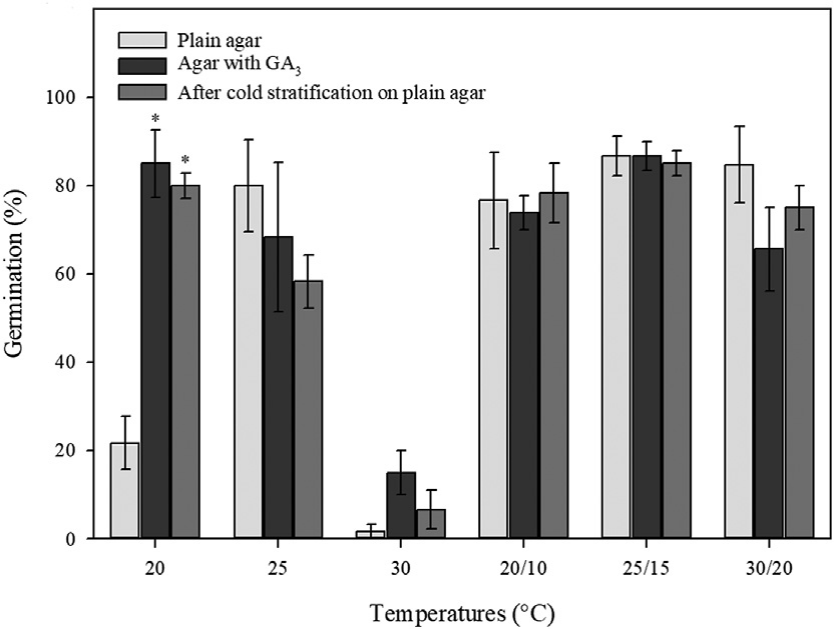
Germination (mean ± s.e.) of freshly matured seeds of *Magnolia sinica* at different temperatures on plain agar or agar with 200 mg/L gibberellic acid (GA_3_). Asterisk (∗) indicates significant difference compared with the germination on plain agar at the same temperature.

Germination occurs after seed swelling and cracking of endotesta along the perimeter. Germination of freshly matured seeds ranged from ca. 1.67% at 30 °C on plain agar to ca. 86.7% at 25/15 °C on plain agar and on agar with GA_3_. GA_3_ and cold stratification promoted germination significantly at 20 °C (Fig. 3). Nearly all (94.1%) of the un-germinated seeds at 20 °C on plain agar remained fresh. In contrast, 50.7% and 97.8% of un-germinated seeds decayed after incubation at 30 °C on plain agar, and on agar with GA_3_ (200 mg/L), respectively. There was evidence too that ungerminated seeds at 25 and 30/20 °C deteriorated in the presence of GA_3_.

### 3.3 Desiccation tolerance and storage behavior

The initial moisture content of freshly matured seeds was 15.23%. The actual MC of seeds dried to the target of 10% MC was 9.27%, and for seeds equilibrated at 15% eRH at 15 °C was ca. 4.85%, very close to the 5% MC target.

**Fig. 4:**
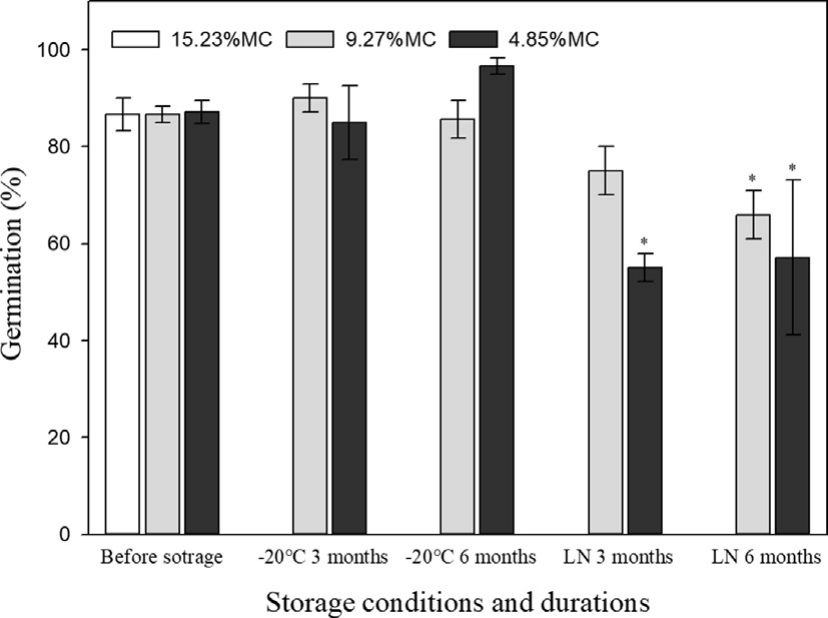
Germination (mean ± s.e.) of *Magnolia sinica* seeds at 25/15 °C on agar with 200 mg/L gibberellic acid (GA_3_) after storage at −20 °C and in liquid nitrogen (LN) for 3 and 6 months with different moisture contents (MC). Asterisk (∗) indicates significant difference compared with the germination of freshly matured seeds.

There was no significant reduction in germination when seeds were dried to either 9.27% or 4.85% MC, nor for seeds at these MCs stored at −20 °C for 3 and 6 months (Fig. 4). A slight but non-significant reduction in germination level was observed for seeds that had been cryopreserved for 3 months at 9.27% MC. However, seeds that were cryopreserved for 3 months with 4.85% MC as well as cryopreserved for 6 months with either 4.85 or 9.27% MC showed a significant reduction in germination (Fig. 4). Total seed viability data was usually slightly higher than the germination data, and only seeds that had been cryopreserved for 3 months at 4.85% MC had a significantly lower viability compared to the freshly matured seeds (Table 1).

**Table 1:** Seed viability (mean ± standard error, %) and germination (%) of excised embryos (in bracket) of *Magnolia sinica* of different moisture contents and storage conditions.

Moisture content (MC)	15.23% MC	9.27% MC	4.85% MC
Before storage	86.67 ± 3.33 (95.7)	93.33 ± 1.67 (100)	87.08 ± 2.39 (62.5∗)
3 months at −20 °C	NA (NA)	95.00 ± 2.89 (78.3)	89.82 ± 5.78 (86.4)
6 months at −20 °C	NA (NA)	90.92 ± 3.80 (58.33∗)	98.24 ± 1.75 (83.33)
3 months at −196 °C	NA (NA)	91.11 ± 3.89 (80.0)	71.14 ± 1.98∗ (87.5)
6 months at −196 °C	NA (NA)	80.35 ± 3.42 (68.00∗)	77.54 ± 12.80 (75.00)

Asterisk (∗) indicates significant difference compared with the data of freshly matured seeds.

### 3.4 Germination of excised embryos

Embryos excised from freshly matured seeds germinated 95.7% *in vitro*. Germination of excised embryos *in vitro* was significantly lower than the control only for seeds dried to 4.85% MC, seeds stored at −20 °C for 6 months at 9.27% MC, and for seeds cryopreserved for 6 months at 9.27% MC (Table 1). Those data were lower than both the germination (Fig. 4) and viability data (Table 1) of the same treatments. Overall, at least 58% of seedlings were obtained by germination of excised embryos *in vitro* after any combination of desiccation and storage condition and time.

## 4 Discussion

### 4.1 Germination

The length of embryos in fresh *Magnolia sinica* seeds is only about one quarter of the length of the seed, indicating the embryo is relatively small at the time of seed dispersal. Similar observations have been made for the embryos of *Magnolia grandiflora* and *Magnolia × soulangeana* Soul.-Bod. (le Page-Degivry, 1970). In the case of *Magnolia champaca* (L.) Baill. ex Pierre (Fernando et al., 2013) and *Magnolia punduana* (Hook. f. & Thomson) Figlar (Iralu and Upadhaya, 2016), the E:S length ratio in fresh seed is 0.21 and 0.13 respectively. *M. sinica* embryo length did not increase during cold stratification but did after transfer to warmer temperatures for germination (Fig. 2). After cold stratification, the E:S ratio decreased a little bit but not significantly. This was probably due to the seed swelling a little bit after imbibition, but the embryo did not grow during this period. Whilst post-cold stratification embryo growth internally is evident at 25 °C in *M. punduana* (Iralu and Upadhaya, 2016), in *M. sinica*, embryo growth was greatest (reaching 0.6 E:S ratio in four weeks) at 25/15 °C and 30/20 °C (i.e., at average temperatures of 20 °C and 25 °C, respectively). Concomitant with embryo growth was the ability of the seed to germinate to ≥80% (Fig. 3), suggesting the need to attain a critical embryo size to enable germination. In *M. champaca* the mean E:S ratio in germinated seeds is 0.61 (Fernando et al., 2013). The possibility for a ‘physical’ threshold for growth has been proposed in many species with endospermic seeds and small embryos, including in the genus *Ribes* (Mattana et al., 2014) and *Conopodium* (Blandino et al., 2019). This could be driven by the need for the embryo to be physically large enough to enable the generation of sufficient thrust to puncture the testa (Fig. 1c). High germination (ca. 80%) is also achieved in *M. sinica* seeds at 20 °C and 20/10 °C. At these temperatures, the E:S is still only about 0.3 four weeks after release from the cold treatment (Fig. 3) and germination occurs later. An E:S ratio of 0.3 is also associated with a later gemination capability of only 60% at 25 °C (Fig. 3). In these cases, the embryo may have grown slowly beyond the recording interval of 4 weeks, also explaining the slower germination. As in *Conopodium majus* (Gouan) Loret (Blandino et al., 2019), *Paeonia corsica* Sieber ex Tausch (Porceddu et al., 2016) and *Aquilegia barbaricina* Arrigoni & E. Nardi (Porceddu et al., 2017), the thermal control of embryo growth in *M. sinica* appears to be very precise, with temperatures close to 25 °C approaching an upper limit.

The temperature window for *Magnolia sinica* seed germination also seems to be relatively narrow, around mean temperatures between 15 °C and 25 °C (Fig. 3). Without prechilling, 20 °C is too cold and 30 °C is too warm.

Contrary to the findings on *M. punduana* (Iralu and Upadhaya, 2016) and *M. champaca* (Fernando et al., 2013), the effects of prechilling and/or GA_3_ on *M. sinica* seed germination is rather limited, i.e., to 20 °C (Fig. 3). We presume, therefore, that this seed lot of *M. sinica* has only a slight degree of dormancy, something that was also not ruled out for some populations of *M. champaca* (Fernando et al., 2013). Consistent with this interpretation is the observation that the benefit to germination under alternating temperatures of 25/15 °C compared with a constant 20 °C does not extend to the 30/20 °C and 25 °C (Fig. 3). Also, after cold stratification, embryos grew faster at those two alternative temperatures (25/15 and 30/20 °C) than at all constant temperatures (Fig. 2). Nonetheless, a small preference for alternating vs. constant temperature could serve as a gap detecting mechanism (Fenner and Thompson, 2005), and seedling establishment may require certain disturbance in the natural environment.

The general pattern of temperature responsiveness is consistent with the species distribution at high altitudes, with the seeds shed in autumn, with some physiological benefit from a period at cool temperatures and then germination maximally efficient at warm but not hot temperatures. Poor germination at 30 °C both with and without GA_3_, and the decaying of a considerable portion of seeds at this temperature indicates that 30 °C not only suppresses germination but also compromises viability. It is likely, therefore, that the ceiling temperature (T_c_) for *Magnolia sinica* seed germination is around 30 °C. A similar depressing effect of 30 °C on germination was found for *M. wilsonii* seeds (Han and Long, 2010b). Consequently, climate warming might have a negative impact on *M. sinica* germination, either during the development on the parent plant or post-harvest (Fernández-Pascual et al., 2019).

As to the type of dormancy, it might be of the non-deep simple MPD kind (Baskin and Baskin, 2014), based on the features of embryo growth and the response of seed to prechilling, temperature and GA_3_. The morphophysiological dormancy (MPD) of *Magnolia sinica* seeds seems not to be of the intermediate complex type, for morphological dormancy (MD) is not broken during cold stratification (i.e., embryos do not grow at low temperatures). As both GA_3_ and 1-month moist chilling promoted germination at 20 °C, the physiological component of dormancy could be non-deep, particularly as embryo growth and germination both occurred at warm temperatures (Fig. 1, Fig. 2). A more complex set of move-along experiments is needed to validate such an interpretation.

### 4.2 Storage

The fresh seeds of *Magnolia sinica* used here were mature enough to be fully desiccation tolerant, as drying to either 9.27% or 4.85% MC had no adverse effect on germination level (Fig. 4). In contrast, *Magnolia ovata* (A. St.-Hil.) Spreng. seeds only survive desiccation to ca. 10% MC when dried relatively slowly using a series of salt solutions with different RHs; fast drying to this MC with silica gel kills the seeds (Jose et al., 2011). Moreover, *M. sinica* seeds survived dry storage for 3 and 6 months at a conventional seed bank temperature of −20 °C (Fig. 4, Table 1). Consequently, seeds of this species appear to have an orthodox storage behavior (Hong and Ellis, 1996). Whilst slow-dried *M. ovata* seeds at 9% MC also survive 90 days at −20 °C, 180 days compromises viability significantly, as does drying to ca. 4.8% MC (Jose et al., 2011). Thus, *M. ovata* seeds appear to be non-orthodox in their seed storage response (Jose et al., 2011). Such a non-orthodox response is quite common in seeds of many Chinese Magnoliaceae species, including *Magnolia baillonii* Pierre (Dent, 1948; Royal Botanical Gardens of Kew, 2019), *M. champaca* (Han, 2008), *M. compressa* Maxim. (Lin and Huang, 1994), *M. duclouxii* (Han, 2008), *M. fulva* (Han, 2008; Han and Long, 2010a; Royal Botanical Gardens of Kew, 2019), *M. laevifolia* (Han, 2008), *M. liliifera* (L.) Baill. (Han and Sun, 2013), *M. sargentiana* Rehder & E.H. Wilson (Tang, 2014), *M. sphaerantha* (Han, 2008), and *M. wilsonii* (Han, 2008; Han and Long, 2010b).

Whilst a non-orthodox seeds storage response means that conventional seed banking is not possible, orthodox seeds of *Magnolia sinica* are potentially bankable for the long term, which is important for the conservation of this critically endangered species. Nonetheless, we also explored opportunities to store the seeds at ultra-low temperature, i.e., cryopreservation. But *M. sinica* seed cryopreservation at 9.3% and 4.9% MC causes a decrease in germination from ca. 90% before storage to about 55–65% germination after 6 months storage, although for the lower MC seed germination seems to remain level between 3- and 6-months storage. Interestingly, both the cut test (71–91%) and germination of excised axes *in vitro* (68–88%) imply that many of the un-germinated seeds could still have been viable (Table 1). The generally higher recovery growth for embryos *in vitro* than for germination after the cryo-storage experiments (cf. Fig. 4 and Table 1) suggests that some limitation of cryopreservation probably resides in the endosperm rather than in the embryo. Similar differences between endosperm and embryo desiccation tolerance and cryopreservation are also known for some other species, e.g. *Calamus manan* Miq. (Royal Botanic Gardens Kew, 2019) and *E. guineensis* (Grout et al., 1983; Ellis et al., 1991). For the seeds, optimization of the cryo techniques is clearly needed. Whilst the *M. sinica* seed MCs used for cryopreservation are around the optima of other species, i.e., 7–14% (Pritchard, 2007), it is possible that plunging the seeds into liquid nitrogen results in cooling that is too fast for such relatively large seeds (Fig. 1). Only with further optimization could cryopreservation be considered for the long-term conservation of this important and endangered plant species with extremely small population (PSESP).

The germination of *Magnolia sinica* embryos *in vitro* is feasible, although the response is evidently a little variable and not always comparable to the germination response. One challenge is the excision of the very small embryo (ca. 800 μm long; Fig. 1d) without damage. Nonetheless, at least 58% seedlings could be obtained; so, this method still has potential as a quick means of viability assessment and could be used to support the development of an *in vitro* multiplication system for magnolia germplasm. Moreover, the ease of growing *M. sinica* embryo from fresh seeds suggests no obvious presence of deep dormancy in the embryo, as earlier noted for isolated embryos of *M. × soulangeana* and *M. grandiflora* (lePage-Degivry, 1970).

In conclusion, the seeds of *Magnolia sinica* have a small embryo, the dormancy status is probably non-deep simple MPD, germinating well at 25/15 °C on plain agar. Moreover, the excised embryo can be germinated *in vitro*. The seed is not only desiccation tolerant but also survives storage over many months under conventional banking and cryo-banking conditions. These findings provide the evidence-base for an accelerated program of conservation and recovery of this highly threatened species.

## Author contributions

L.L., X-J.H. and X-Y.Y. initiated and designed this study, L.C. collected the seeds and performed the morphological and anatomical study, L.F. and X-J.H. performed the germination, desiccation and cold storage study, J-C.M. and L.L. performed the cryopreservation and *in vitro* germination of excised embryos. X-J.H., L.L., L.C. and X-Y.Y. wrote the first draft of the manuscript. All authors read and revised the manuscript, all authors approved the final manuscript.

## Declaration of competing interest

The authors declare that they have no known competing financial interests or personal relationships that could have appeared to influence the work reported in this paper.

## References

[bib1] Ballesteros D., Hill L.M., Lynch R.T. (2019). Longevity of preserved germplasm: the temperature dependency of aging reactions in glassy matrices of dried fern spores. Plant Cell Physiol..

[bib2] Baskin C.C., Baskin J.M. (2014).

[bib3] Blandino C., Fernández-Pascual E., Marin M. (2019). Seed ecology of the geophyte *Conopodium majus* (Apiaceae), indicator species of ancient woodland understories and oligotrophic meadows. Plant Biol..

[bib4] Campbell M. (1980).

[bib5] Dent T.V. (1948). Indian Forest Records Silviculture. Delhi.

[bib6] Ellis R.H., Hong T.D., Roberts E.H. (1990). An intermediate category of seed storage behaviour?. I. Coffee. J. Exp. Bot..

[bib7] Ellis R.H., Hong T.D., Soetisna U. (1991). Seed storage behaviour in *Elaeis guineensis*. Seed Sci. Res..

[bib8] Fenner M., Thompson K. (2005).

[bib9] Fernández-Pascual E., Mattana E., Pritchard H.W. (2019). Seeds of future past: climate change and the thermal memory of plant reproductive traits. Biol. Rev..

[bib10] Fernando M.T.R., Jayasuriya K.M.G.G., Walck J.L. (2013). Identifying dormancy class and storage behaviour of champak (*Magnolia champaca*) seeds, an important tropical timber tree. J. Natl. Sci. Found. Sri Lanka.

[bib11] Fetouh M.I., Hassan F.A. (2014). Seed germination criteria and seedling characteristics of *Magnolia grandiflora* L. trees after cold stratification treatments. Int. J. Curr. Microbiol. App. Sci..

[bib12] Grout B.W.W., Shelton K., Prichard H.W. (1983). Orthodox behaviour of oil palm seed and cryopreservation of the excised embryo for genetic conservation. Ann. Bot..

[bib13] Han C.Y. (2008).

[bib14] Han C.Y., Long C.L. (2010). Dormancy, germination and storage of *Magnolia ingrata* seeds. Seed Sci. Technol..

[bib15] Han C.Y., Long C.L. (2010). Seed dormancy, germination and storage behavior of *Magnolia wilsonii* (Magnoliaceae), an endangered plant in China. Acta Bot. Yunnanica.

[bib16] Han C.Y., Sun W.B. (2013). Seed storage behaviour of *Magnolia odoratissima*. Seed Sci. Technol..

[bib17] Hong T.D., Ellis R.H., Engels J.M.M., Toll J. (1996). IPGRI Technical Bulletin No. 1.

[bib18] ISTA (1996). International rules for seed testing. Seed Sci. Technol..

[bib19] Iralu V., Upadhaya K. (2016). Dormancy, storability and germination of seeds of *Magnolia punduana* (Magnoliaceae). Botany.

[bib20] Jose A.C., da Silva A.A., Davide A.C. (2011). Effects of drying rate and storage time on *Magnolia ovata* Spreng. seed viability. Seed Sci. Technol..

[bib21] le Page-Degivry M.T. (1970). Seed dormancy associated with embryo immaturity: contribution for the study of *Magnolia soulangeana* Soul. Bod. and *Magnolia grandiflora* L. by means of *in vitro* culture. Planta.

[bib22] Li D.-Z., Pritchard H.W. (2009). The science and economics of *ex situ* plant conservation. Trends Plant Sci..

[bib23] Lin T.P., Huang N.H. (1994). The relationship between carbohydrate composition of some tree seeds and their longevity. J. Exp. Bot..

[bib24] Lloyd G., McCown B. (1980). Commercially-feasible micropropagation of mountain laurel, *Kalmia latifolia*, by use of shoot tip culture. Int. Plant Propagators’ Soc. Proc..

[bib25] Ma Y., Chen G., Grumbine R.E. (2013). Conserving plant species with extremely small populations (PSESP) in China. Biodivers. Conserv..

[bib26] Mattana E., Stuppy W.H., Fraser R. (2014). Dependency of seed dormancy types on embryo traits and environmental conditions in *Ribes* species. Plant Biol..

[bib27] Ministry of Environmental Protection of the People’s Republic of China and Chinese Academy of Sciences (2013). http://www.mee.gov.cn/gkml/hbb/bgg/201309/W020130917614244055331.pdf.

[bib28] Porceddu M., Mattana E., Pritchard H.W. (2016). Sequential temperature control of multi-phasic dormancy release and germination of *Paeonia corsica* seeds. J. Plant Ecol..

[bib29] Porceddu M., Mattana E., Pritchard H.W. (2017). Dissecting seed dormancy and germination in *Aquilegia barbaricina*, through thermal kinetics of embryo growth. Plant Biol..

[bib30] Pritchard H.W., JG D., Stacey G. (2007). Cryopreservation and Freeze-Drying Protocols.

[bib31] R Core Team (2019). http://www.R-project.org/.

[bib32] Royal Botanic Gardens Kew (2019).

[bib33] Shu X., Yang X., Yang Z. (2012). Variation in seed and seedling traits among fifteen Chinese provenances of *Magnolia officinalis*. Not. Bot. Horti Agrobot. Cluj-Napoca.

[bib34] Tang A.J. (2014). Seed dormancy and storage behavior of *Magnolia sargentiana* endemic to China. Plant Physiol. J..

[bib35] Wade E.M., Nadarajan J., Yang X. (2016). Plant species with extremely small populations (PSESP) in China: a seed and spore biology perspective. Plant Divers.

[bib36] Wang B., Ma Y., Chen G. (2016). Rescuing *Magnolia sinica* (Magnoliaceae), a critically endangered species endemic to Yunnan, China. Oryx.

[bib37] Xia N., Liu Y., Nooteboom H.P. (2008). Flora of China. Science Press, Beijing.

[bib38] Yang J., Cai L., Liu D. (2020). China's conservation program on plant species with extremely small populations (PSESP): progress and perspectives. Biol. Conserv..

[bib39] Zheng Y.L., Sun W.B. (2009). Seed germination of huagaimu, a critically endangered plant endemic to southeastern Yunnan, China. Horttechnology.

